# Risk Factors for Culling, Sales and Deaths in New Zealand Dairy Goat Herds, 2000–2009

**DOI:** 10.3389/fvets.2017.00191

**Published:** 2017-11-10

**Authors:** Milan Gautam, Mark A. Stevenson, Nicolas Lopez-Villalobos, Victoria McLean

**Affiliations:** ^1^Institute of Veterinary, Animal and Biomedical Sciences, Massey University, Palmerston North, New Zealand; ^2^Faculty of Veterinary and Agricultural Sciences, The University of Melbourne, Parkville, VIC, Australia; ^3^Dairy Goat Co-Operative, Hamilton, New Zealand

**Keywords:** epidemiology, dairy goats, length of productive life, survival analysis, Cox proportional hazards regression

## Abstract

The aim of this study was to identify risk factors for culling, sales and deaths in intensively managed dairy goat herds in New Zealand. A data set provided by the New Zealand Dairy Goat Cooperative (*n* = 13,197 does) was analyzed using a Cox proportional hazard model. The outcome of interest was length of productive life (LPL), defined as the number of days from the date of second kidding to the date of removal from the herd or the date on which follow-up was terminated, whichever occurred first. Milk solids yield in the first lactation (MSL1) as a predictor of LPL was parameterized in the model as a penalized spline term. To account for MSL1 violating the proportional hazards assumption of the Cox model, LPL was divided into two intervals: T1 (less than or equal to 730 days from the date of second kidding) and T2 (greater than 730 days from the date of second kidding). MSL1 was then included in the model as a time-dependent covariate. A frailty term was included in the model to account for unmeasured, herd-level effects on LPL. During T1, the daily hazard of removal for does that produced 80 kg milk solids in the first lactation was 0.84 (95% CI 0.58–1.23) times the daily hazard of removal for does that produced 30 kg milk solids in the first lactation. During T2, the daily hazard of removal for does that produced 80 kg milk solids in the first lactation was 1.44 (95% CI 0.79–2.65) times the daily hazard of removal for does that produced 30 kg milk solids in the first lactation. We conclude that involuntary losses may be avoided if high MSL1 yielding does are preferentially managed from 2 years beyond the date of second kidding.

## Introduction

In farmed animal production systems (e.g., dairy, beef cattle, pig, and dairy goat farms) a long, productive life of individual production units is an essential prerequisite for economic efficiency (1). In dairy systems, longevity is defined as the interval between delivery of the first offspring and the date of removal from the herd (2). Increasing the longevity of dairy animals is desirable because it means that the cost of rearing replacements is amortized over a longer period of income production. Since longevity is a desirable quality in production animals (3), it is important to have an understanding of factors influencing the same. Very little work has been done in this area of the dairy goat industry, and an understanding of risk factors for culling, sales and deaths in dairy goats is limited.

In New Zealand, the number of dairy goat herds is small relative to the number of dairy cow herds, and a key industry focus is on the production of infant formula (4). Typically, does are housed indoors in open sided free stall barns and are fed fresh-cut pasture. Approximately two-thirds of the commercial dairy goat farms are concentrated in the Waikato region, in the upper North Island. Purebred and crossbred Saanens are the predominant breeds, but other breeds such as Toggenburgs and Alpines are common (4). At the time of writing, there were 69 herds registered with the New Zealand Dairy Goat Cooperative (NZDGC), a farmer-owned cooperative, each with around 700 milking does per herd, on average.

A better understanding of the various risk factors for removal can be used to enhance longevity in dairy animals. With this knowledge, it is possible to identify characteristics that can serve as early indicators of culling and, depending upon how strong the effect of a particular risk factor on removal is, it is possible to plan in advance the best time to remove an animal from the herd when it is still profitable to do so or at least incur minimal loss. Survival analysis is a commonly used technique to quantify longevity in domestic animals (5, 6). Using this technique, the association between risk factors and culling can be examined in relation to their effect on the length of productive life (LPL) instead of simply describing the relationship in terms of risk (5). In survival analysis, a quantity termed “hazard” is modeled instead of longevity itself (7). Hazard represents the instantaneous probability that an animal is removed at a given time, given that it is still present up to that time. Since it is the hazard that is modeled and not longevity, it is possible to use data from animals that have not yet been removed from the herd (as censored observations) as well as those that have been removed (7).

Although a number of studies have been carried out to identify risk factors for removal in dairy cows (5, 8, 9), the number of similar studies in dairy goats is limited (1, 10) and, to the best of our knowledge, none have been conducted in a New Zealand context. To address this knowledge gap, the aim of this study was to identify factors that influence the risk of removal in commercial dairy goat herds in New Zealand (11). This knowledge will allow managers of dairy goat herds take a more planned approach to culling: either to remove does at higher risk of removal at a time when it is economic to do so, or to preferentially manage profitable animals if it is known that they are at greater risk of removal compared to their herd mates.

## Materials and Methods

### Study Population and Data Collection

The data for this study were obtained from the NZDGC. Since the total number of dairy goat herds in New Zealand is relatively small, we assumed the dairy goat herds affiliated with NZDGC provided an accurate reflection of commercial dairy goat farming in New Zealand. Although the complete data set was comprised of records for a total of 48,699 animals (including those with birth dates as early as August 1983 and production records up to December 2009), only those born on or after 1st January 2000 were used in the analyses presented in this paper. This restriction was applied because a large proportion of animals born prior to 1st January 2000 had missing observations, particularly those related to total lactation length and milk, fat, and protein yields.

**Figure 1:**
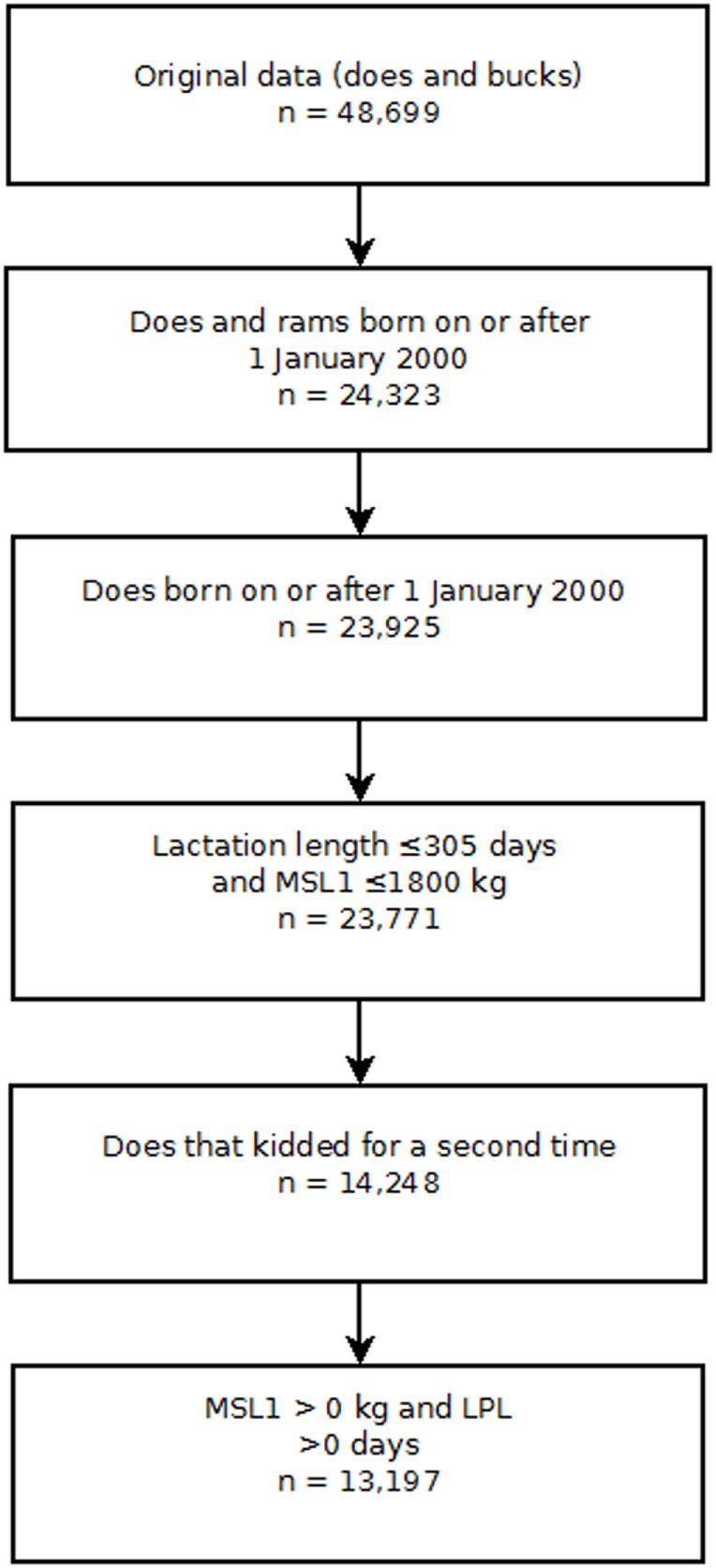
Risk factors for culling, sales and deaths in New Zealand dairy goat herds, 2000–2009. Flow chart showing the exclusion criteria used to select individual doe records for analysis in this study. Key: MSL1 first lactation milk solids yield (kilograms); LPL, length of productive life.

Several exclusion criteria were applied to the NZDGC data (Figure 1). Bucks were excluded from the analyses. A doe had to complete her first lactation and then kid for a second time to be included in the data set so that the correct temporal sequence between first lactation milk solids yield (MSL1) and LPL was ensured. Finally, records were screened and limited to does having a first lactation length between 0 and 305 days and/or a first lactation total milk solids yield of less than or equal to 1,800 kg. Lactations of greater than 305 days and total lactation yields of more than 1,800 kg milk solids were deemed implausible. Finally, does for which the first lactation fat and protein yields were recorded as 0 were excluded from the analyses. Does were followed until 31st December 2009 or the date on which they were removed from the herd, whichever occurred first.

Herds registered with the NZDGC record data for individual animals including the date of birth, the unique animal identifier, breed, parity date(s), and the date and reasons for removal from the herd (if applicable). Herd managers record details of individual animals into paper diaries or, more rarely in the case of dairy goats, into dedicated herd health software. This information is then sent to the national milk recording authority, Livestock Improvement Corporation (LIC) who merge these details with test day milk yields measured at roughly 60-day intervals throughout the lactation. Animal biographical and production data recorded in the central database of LIC are then transferred to NZDGC in digital format. This information is used by NZDGC for genetic evaluation of individual animal (12). Estimated breeding values for milk, fat, protein, and milk solids (fat and protein) obtained from genetic evaluations are reported to the NZDGC and each herd manager receives an individual report with the genetic evaluation of his/her animals.

The outcome of interest in this study was LPL, defined as the difference in time (days) between the date of second kidding and the date of removal from the herd. In the context of this study, we use the term “removal” to refer to animals that leave the herd as either culled animals, sales, or deaths. For does that were still in the herd at the termination of the study (censored observations), LPL was quantified as the time between the date of second kidding and 31st December 2009.

### Model Building

#### Selection of Explanatory Variables

The total yields of milk protein and milk fat from each animal in the first lactation were added to create a single variable called first lactation milk solids yield (MSL1).

Based on the reported breed composition of the sire and dam the breed of each animal was recorded in 16th for the following breeds: Saanen, Toggenburg, Nubian, Alpine, and “unknown.” From these fractions (the total of which sum to one), the proportion of each breed was calculated. For instance, the breed composition of a doe with pedigree values 8, 4, 0, 0, 4 for Saanen, Toggenburg, Nubian, Alpine, and unknown (respectively) would be 50% Saanen, 25% Toggenburg, 0% Nubian, 0% Alpine, and 25% unknown. Given the several possible combinations of crossbreds, it was decided that the percentage of each breed would be forced into the model as a series of continuous variables to avoid any ambiguity created by breed defined as a categorical variable. The recorded parentage details for all does were not available. Where parentage details were not available, breed fractions were estimated by the herd manager.

#### Bivariate Analyses

Since all the explanatory variables in our study were continuously distributed, they were categorized into quartiles. The Kaplan–Meier technique (13) was then used to quantify LPL of does within each quartile. The log rank statistic was used to test the homogeneity of survivorship between quartile groups. Those explanatory variables that showed an association with LPL (that is, a difference in the Kaplan–Meier survival curves that was significant at *P* < 0.20) were selected for inclusion in the multivariate analyses.

#### Multivariable Analyses

Factors influencing LPL were quantified using a Cox proportional hazard model (14). Here, the hazard of removal at time *t* can be expressed as:

(1) $$H(t,x)=h_{0}(t){\text{exp}}^{\beta_{1}x_{1i}+\beta_{2}x_{2i}+\ldots +\beta_{k}x_{ki}}.$$

Equation 1 shows the hazard of an event at time *t* is the product of *h*_0_(*t*) and ${\text{exp}}^{\beta_{1}x_{1i}+\beta_{2}x_{2i}+\ldots +\beta_{k}x_{ki}}$. The first of these quantities, *h*_0_(*t*), is called the baseline hazard function and includes a time component *t*, representing how the hazard of removal changes as a function of time. The remaining quantity ${\text{exp}}^{\beta_{1}x_{1i}+\beta_{2}x_{2i}+\ldots +\beta_{k}x_{ki}}$ is the exponential of the linear sum of a series of *k* explanatory variables. This quantity represents how the baseline hazard function is modified in response to a given set of explanatory variables. In contrast to the baseline hazard function, the set of explanatory variables does not involve a time component (15).

A key assumption of the Cox model is that of proportionality of hazards. According to this assumption, the effect of an explanatory variable on the outcome of interest does not change over time, i.e., the hazards for each level of an explanatory variable must be proportional at all times. In situations where this assumption is violated, modifications such as stratified analyses or inclusion of time-dependent covariates are necessary (16).

Model development was carried out using the contributed survival package (17) implemented in R version 3.3.3 (18). To start, a saturated Cox model was run including all explanatory variables identified as influencing LPL at the bivariate level. Explanatory variables that were not statistically significant were removed from the model one at a time, beginning with the least significant, until the estimated regression coefficients for all explanatory variables retained were significant at an alpha level of less than 0.05. Explanatory variables that were excluded at the initial screening stage were tested for inclusion in the final model and were retained in the model if their inclusion changed any of the estimated regression coefficients by more than 20%. Biologically plausible two-way interactions were between explanatory variables were assessed.

#### Checking the Scale of Continuous Covariates

A key assumption in including MSL1 into the model as a continuous variable was that the relationship between MSL1 and log hazard was linear. To test this assumption, MSL1 was categorized into quartiles and the regression coefficient for each quartile plotted as function of the midpoint of each quartile group. Since the line connecting the four midpoints was not linear, we concluded that MSL1 was not linear in its log hazard. Based on these findings, a penalized spline term was used to account for the non-linear association between MSL1 and LPL.

#### Testing the Proportional Hazard Assumption

To verify that the proportional hazards assumption of the Cox model was valid a plot of the scaled Schoenfeld residuals from the model as a function of time was constructed. In a model where the proportional hazards assumption holds the Schoenfeld residuals should be scattered around 0. We calculated the Pearson product–moment correlation between the scaled Schoenfeld residuals and time and the hypothesis of no correlation between the two variables was assessed using a χ^2^ test statistic. From these analyses, we concluded that MSL1 violated the proportional hazards assumption. To account for non-proportionality of hazards, we divided LPL into two intervals: less than or equal to 730 days (referred to as T1 in the remainder of this paper) and greater than 730 days (T2). The decision to use 730 days was semi-arbitrary and was selected because, being equivalent to 2 years, it approximated median LPL in this population. This division allowed us to quantify the effect of MSL1 separately for each period [less than or equal to 730 days (T1) and greater than 730 days (T2)]. The technique of dividing the time component into intervals to investigate the time-dependent effect of covariates is called a piecewise Cox proportional hazards model or a step function proportional hazards model.

#### Final Model

In addition to the terms to allow for the interaction between time and penalized MSL1, our final model included herd as a random effect, otherwise known as a frailty term.

## Results

The final data set was comprised of 23,771 does with a birth date greater than or equal to 1st January 2000. Of this group, 14,248 does completed their first lactation and kidded for the second time. Further screening of the production data and removal of implausible records reduced the final data set to comprised 13,197 does from 38 herds (Figure 1). Of this group, 5,386 animals were removed during the follow-up period and the remaining 7,811 animals that were recorded as being alive in the herd on 31st December 2009 were treated as censored observations. Descriptive statistics of the study population are presented in Table 1.

**Table 1:** Risk factors for culling, sales and deaths in New Zealand dairy goat herds, 2000–2009.

Outcome	*n*	Mean	SD	Median	Q1; Q3
L1 fat yield (kg)	13,197	16	8	16	10; 21
L1 protein yield (kg)	13,197	14	7	14	9; 18
L1 milk solids (kg)	13,197	30	15	29	19; 40
Age at first kidding (days)	13,197	580	421	390	369; 669
LPL (days)	5,386^a^	763	547	663	327; 1,084
Age at removal (days)	5,386^a^	1,644	596	1,500	1,142; 2,026
Number of lactations	5,386^a^	3	1.4	3	2; 4

*Descriptive statistics of first lactation production outcomes, age at first kidding, LPL, age at removal and total number of lactations*.

*L1, lactation 1; LPL, length of productive life; Q1, first quantile; Q3, third quantile*.

*^a^Uncensored does only*.

Inclusion of terms for breed in the Cox proportional hazards model was not statistically significant. Biologically plausible two-way interactions were tested and none were significant at an alpha level of 0.05.

**Figure 2:**
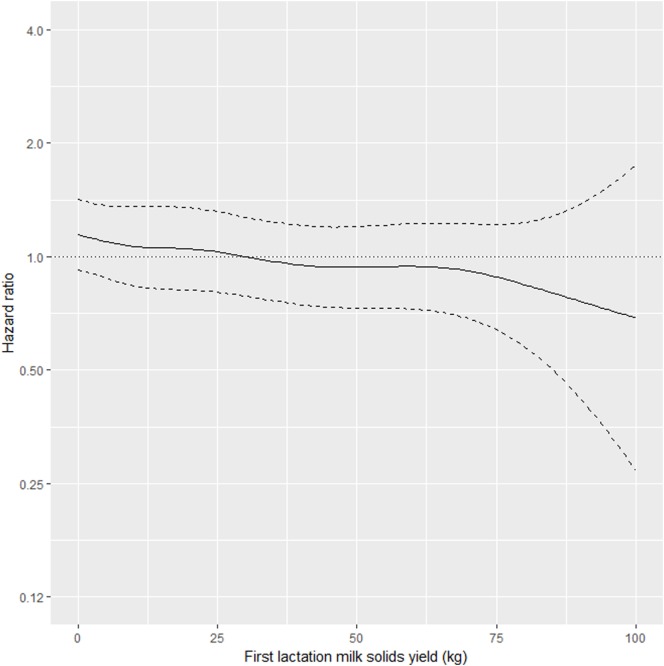
Risk factors for culling, sales and deaths in New Zealand dairy goat herds, 2000–2009. Line plot showing, for the interval 0–730 days from the date of second kidding, the hazard ratio for removal as a function of first lactation milk solids yield (based on the model presented in Table 2). The dashed lines represent 95% confidence intervals around the point estimates of the hazard ratio. In the above plot, the reference category was a doe producing 30 kg milk solids in the first lactation. A doe producing 80 kg milk solids in the first lactation had 0.84 (95% CI 0.58–1.23) times the daily hazard of removal compared with a doe that produced 30 kg milk solids in the first lactation.

**Figure 3:**
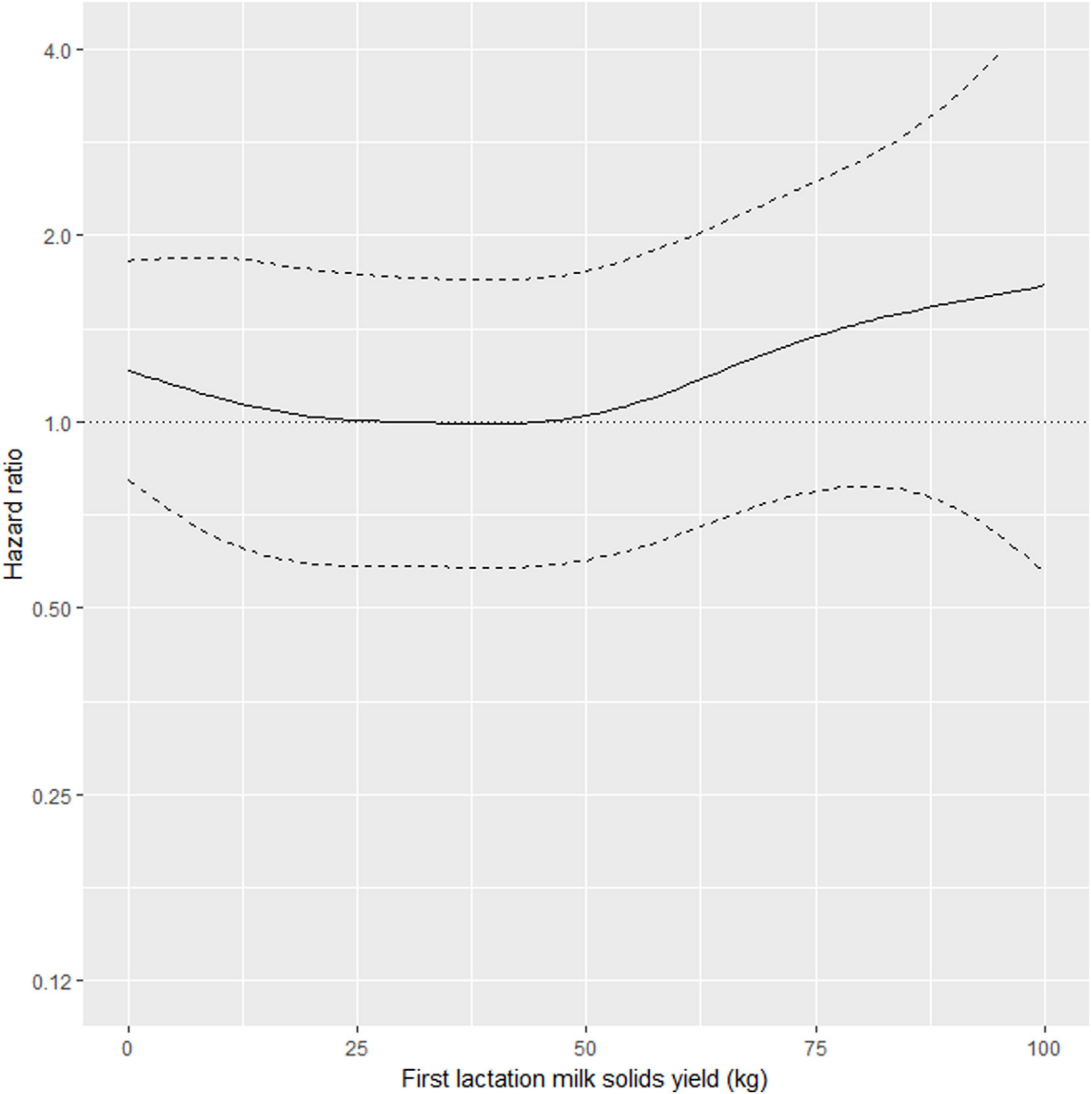
Risk factors for culling, sales and deaths in New Zealand dairy goat herds, 2000–2009. Line plot showing, for the interval greater than 730 days from the date of second kidding, the hazard ratio for removal as a function of MSL1 (based on the model presented in Table 2). The dashed lines represent 95% confidence intervals around the point estimates of the hazard ratio. In the above plot, the reference category was a doe producing 30 kg milk solids in the first lactation. A doe producing 80 kg milk solids in the first lactation had 1.44 (95% CI 0.79–2.65) times the daily hazard of removal compared with a doe that produced 30 kg milk solids in the first lactation.

As shown in Table 2, the interaction between MSL1 and time was significant for T1, but was not statistically significant for T2. During T1, the hazard of removal for does that produced 80 kg milk solids in the first lactation was 0.84 (95% CI 0.58–1.23) times the daily hazard of removal for does that produced 30 kg milk solids in the first lactation (Figure 2). During T2 (730 days after the date of second kidding), high producing MSL1 does had a higher daily hazard of removal compared to average producing herd mates: a doe producing 80 kg milk solids in the first lactation had 1.44 (95% CI 0.79–2.65) times the daily hazard of removal compared with does that produced 30 kg milk solids in the first lactation (Figure 3). These results show that relatively high levels of MSL1 production had no strong association with daily hazard of removal during the early phase of productive life, however, as LPL progressed, does with higher MSL1 yields were at greater risk of removal.

**Table 2:** Risk factors for culling, sales and deaths in New Zealand dairy goat herds, 2000–2009.

Variable	Coefficient (SE)	Chi square	df	P
**MSL1 × T1**				
Linear	−0.0033 (0.0014)	5.31	1.0	0.021
Non-linear	–	1.68	3.0	0.650
**MSL1 × T2**				
Linear	0.0014 (0.0016)	1.00	1.0	0.360
Non-linear		3.05	3.0	0.030
Herd-level random effect	–	2,358.74	13.60	0.000

*Regression coefficients of factors influencing risk of culling in dairy goats from the final piecewise Cox model*.

*MSL1, first lactation milk solids yield (kilogram); T1, 0–730 days from the date of second kidding; T2, greater than 730 days from the date of second kidding*.

## Discussion

We used a piece-wise Cox proportional hazards model, to quantify the effect of MSL1 on LPL in dairy goats that completed their first lactation and kidded a second time. To the best of our knowledge, this is the first study of its kind to evaluate the effect of a time-dependent covariate on longevity in dairy goats.

Although the results presented in this study are based on data which were not originally collected for the purpose of this study, consent to use and analyze the data was obtained from NZDGC before the start of the study and results were presented to NZDGC stakeholders. A possible limitation of our study was selection bias in that the herds used for these analyses were those that participated in herd testing programs and were, therefore, likely to be a more intensively managed subset of dairy goat herds compared with the general population of New Zealand dairy goat herds. A second limitation was that we could not investigate the effect of specific diseases or disease categories on longevity. There were two reasons for this: (1) we had no reassurance that disease case definitions were used consistently over time and across each of the herds that took part in the study; and (2) does were removed for a wide range of reasons resulting in relatively low numbers of animals in each category. When studying factors influencing LPL in production animals, it is desirable to identify risk factors for specific removal reasons (e.g., reproductive failure, udder health, lameness) as opposed to considering all removals as a single group. Failure to do so is likely to mask some of the more subtle influences on longevity. As a prerequisite for being able to examine specific reasons for removal, it is necessary that removal reasons are recorded accurately and consistently across herds and over time.

Our results show that in the first 2 years after the date of second kidding, there was an inverse association between MSL1 yields and the daily hazard of removal (Figure 2). Does with higher MSL1 yields had lower daily hazards of removal compared with average producing herd mates. This trend reversed beyond 2 years from the date of second kidding (Figure 3) with high MSL1 yields having a higher daily hazard of removal compared with average producing herd mates. We believe these results provide useful information for the management of dairy goat herds. As high producers get older, herd managers need to take special steps to ensure that this group of animals is managed in such a way to minimize the impact of factors that could influence removal risk. For example, a herd manager might elect to run his/her high MSL1 producers as a separate mob and to provide preferential feeding, housing, and milking management.

A search of the literature did not identify any previous studies that investigated the association between first lactation milk solids yield and longevity in dairy goats. Even in dairy cattle, the number of studies that have examined the association between first lactation milk yield and longevity is limited (19–22). It has been shown that mean daily yield of milk in the first lactation of a cow is an early indicator of lifetime yield (21–23). While the total lifetime yield or daily milk yield in animals in subsequent lactations can be expected to be high in animals that produce more milk in the first lactation, overall reproductive performance decreases (22). Animals producing high amounts of milk in the first lactation are subject to a greater level of metabolic stress as a result of negative energy balance (20), which consequently leads to impaired fertility (24). Since we investigated the effect of MSL1 on LPL instead of first lactation milk yield and our study involved dairy goats, it is not possible to directly extrapolate the results of the above cow-based research to our study. Nevertheless, it is biologically plausible to assume that high yields of milk solids in first lactation would have a negative impact on the energy balance of dairy animals regardless of species. However, with good management, this negative effect may be unapparent for a reasonable period of time which, in our case, was approximately 2 years after the date of second kidding.

In this study, the effect of MSL1 on LPL was investigated using a model that included herd as a random effect (frailty) term. A frailty term is a continuous variable that quantifies the unobserved heterogeneity for groups of individuals such as those in families, classes, schools, or herds (25). Frailty terms are important because they provide a means for accounting for heterogeneity (i.e., “clustering”) in outcome risk that arises from individuals within a cluster being more similar than individuals selected at random from the general population. Since variations in management practices among herds can be expected, the use of herd level effect as a frailty term is a standard practice in epidemiological studies that quantify risk factors for given outcomes in domestic, farmed animal populations (26). The significance of the herd-level effect term in the model indicates that the hazard of removal as a function of LPL varied across herds. We propose that studies comparing herds with upper quartile frailty terms with those with lower quartile frailty terms may be useful to identify specific herd-level factors that are influential determinants of LPL. For example, a cross-sectional questionnaire survey can be designed to investigate various aspects of management such as nutrition, veterinary care, breeding practices, and milking practices in these two categories of farms and the data used to analyze differences between “low risk” and “high risk” herds in terms of survival.

In general, where heterogeneity is an unavoidable feature of the population under investigation, researchers should take into account the existence of dissimilarities among groups to avoid errors during analysis. By failing to acknowledge such heterogeneity, a researcher is more likely to make Type I error, which means he/she is likely to report a false association between explanatory and outcome variables when there is none. Interestingly, the protective effect of high MSL1 on the hazard of removal during T1 was evident only after the effect of herd was accounted-for in the model as a frailty term. When herd-level effects were not controlled-for, high MSL1 in L1 was positively associated with an increase in the risk of removal.

Several studies conducted on dairy cows have studied animal traits affecting LPL. Since longevity usually refers to the time between the first parity of an animal and its removal from the herd, it is not possible to get a direct measure of longevity for all animals, particularly those that are younger (6). However, with the use of survival analysis, such issues can be accounted-for because the technique uses information from all animals used in the study regardless of their culling status at the end of the study. Since we were interested to find out if MSL1 was associated with longevity, we defined longevity as the number of days between the date of second kidding and the date of removal from the herd. In this way, we could be sure that the explanatory variable (MSL1) preceded the study outcome (LPL), ensuring the correct temporal sequence between cause and effect.

## Conclusion

This study identified a time varying effect of MSL1 on removal in New Zealand dairy goats. We found that does with high MSL1 yields had a lower risk of removal during the first 2 years following the second kidding compared with compared with their average producing herd mates. Beyond 2 years following the second kidding, does with high MSL1 yields had a relatively high hazard of removal compared with their average producing herd mates. We conclude that involuntary losses may be avoided if high MSL1 yielding does are preferentially managed from 2 years beyond the date of second kidding.

The data and analyses presented in this paper are based on the first author’s thesis presented as partial fulfillment of the requirements for the degree of Master of Veterinary Studies at Massey University, New Zealand.

## Author Contributions

Study conception and design and critical revision: MG, MS, NL-V, and VM. Acquisition of data: NL-V and VM. Analysis and interpretation of data, and drafting of manuscript: MG and MS.

## Conflict of Interest Statement

The authors declare that the research was conducted in the absence of any commercial or financial relationships that could be construed as a potential conflict of interest. The reviewer CP and handling editor declared their shared affiliation.
